# H3K27me3 of Rnf19a promotes neuroinflammatory response during Japanese encephalitis virus infection

**DOI:** 10.1186/s12974-023-02852-4

**Published:** 2023-07-21

**Authors:** Shuo Zhu, Mengying Tao, Yunchuan Li, Xugang Wang, Zikai Zhao, Yixin Liu, Qi Li, Qiuyan Li, Yanbo Lu, Youhui Si, Shengbo Cao, Jing Ye

**Affiliations:** 1 National Key Laboratory of Agricultural Microbiology, Huazhong Agricultural University, Wuhan, 430070 Hubei China; 2 Laboratory of Animal Virology, College of Veterinary Medicine, Huazhong Agricultural University, Wuhan, Hubei China; 3 The Cooperative Innovation Center for Sustainable Pig Production, Huazhong Agricultural University, Wuhan, Hubei China

**Keywords:** Japanese encephalitis virus, H3K27me3, Rnf19a, Neuroinflammation

## Abstract

Histone methylation is an important epigenetic modification that affects various biological processes, including the inflammatory response. In this study, we found that infection with Japanese encephalitis virus (JEV) leads to an increase in H3K27me3 in BV2 microglial cell line, primary mouse microglia and mouse brain. Inhibition of H3K27me3 modification through EZH2 knockdown and treatment with EZH2 inhibitor significantly reduces the production of pro-inflammatory cytokines during JEV infection, which suggests that H3K27me3 modification plays a crucial role in the neuroinflammatory response caused by JEV infection. The chromatin immunoprecipitation-sequencing (ChIP-sequencing) assay revealed an increase in H3K27me3 modification of E3 ubiquitin ligases Rnf19a following JEV infection, which leads to downregulation of Rnf19a expression. Furthermore, the results showed that Rnf19a negatively regulates the neuroinflammatory response induced by JEV. This is achieved through the degradation of RIG-I by mediating its ubiquitination. In conclusion, our findings reveal a novel mechanism by which JEV triggers extensive neuroinflammation from an epigenetic perspective.

**Supplementary Information:**

The online version contains supplementary material available at 10.1186/s12974-023-02852-4.

## Background

Japanese encephalitis virus (JEV), belonging to the genus *Flavivirus* in the family *Flaviviridae*, is a zoonotic mosquito-borne pathogen that causes an acute inflammatory disease in brain [1]. The genomic RNA of JEV acts as a functional mRNA, producing a polyprotein precursor that undergoes processing by both host and viral proteases to yield three structural proteins (C, prM and E) and seven non-structural proteins (NS1, NS2a, NS2b, NS3, NS4 and NS5) [2]. Targeting the central nervous system (CNS) and triggering an overactive inflammatory response in CNS by JEV has been known as the main cause of the disease [3, 4]. Microglial cells, which are the resident macrophages and the primary effector in immune defense of the CNS [5, 6], become activated in response to JEV infection and mediate inflammatory processes within the CNS [7, 8]. Activation of microglia is considered a crucial defense mechanism to safeguard neurons against invading microorganisms [3]. However, uncontrolled overactivation of microglia can lead to neuronal cell death by producing pro-inflammatory mediators [4, 9], such as interleukin 6 (IL-6), tumor necrosis factor (TNF-α) and C–C motif chemokine ligand 5 (CCL5), which cause toxic effects and promote the recruitment of peripheral immune cells to the CNS [4, 10, 11]. As established by previous studies, the activation of microglia is considered as the key component leading to JEV-induced neuroinflammation [12–15]. However, how JEV cause the excessive inflammatory response in microglia has not been fully understood yet.

Epigenetics modifications, such as histone modifications, DNA methylation and non-coding RNAs, as well as the enzymatic systems that regulate those modifications, have been implicated in regulating cell phenotype and gene expression changes [16, 17]. Histone modifications are essential for regulating gene expression through the post-translational modifications including acetylation, methylation, ubiquitination, sumoylation and phosphorylation [18, 19], among which, methylation of histones occurs primarily on both lysine (K) and arginine (R) residues, leading to either gene activation or repression depending on the location and the specific residue modified [19–22]. The tri-methylation of histone H3 lysine 27 (H3K27me3) catalyzed by histone methyltransferase (HMT) enhancer of zeste homolog-2 (EZH2) is generally associated with transcriptional repression [23, 24]. Recent studies have shown that EZH2 plays a crucial role in the development and progression of inflammatory diseases by mediating H3K27me3 [25, 26]. For instance, EZH2-mediated H3K27me3 was found to play an important role in aberrant cancer gene silencing, as well as in development of experimental autoimmune encephalomyelitis and intestinal inflammation [27–29]. Moreover, the potential use of EZH2 inhibitors in inflammatory diseases has been widely investigated. For example, inhibition of EZH2 was shown to decrease the expression of pro-inflammatory genes such as cytokine signaling 3 (Socs3) in microglial cells and diminishes the activation of neonatal microglia [16, 29].

While it is known that EZH2-mediated histone modifications play a role in various inflammatory diseases, the specific impact of H3K27me3 on the neuroinflammatory response induced by JEV remains unclear. Here, we found that H3K27me3 positively regulates JEV-induced inflammation in microglia via leading to the transcriptional repression of ring finger protein 19A (Rnf19a) which can inhibit the inflammatory response by degradation of retinoic acid-inducible gene I (RIG-I). These findings may provide a new insight into the mechanism of JEV-induced overactivation of neuroinflammation from an epigenetic perspective and potential therapeutic target for Japanese encephalitis.

## Methods

### Cell and virus

BV2 cells (mouse microglial cell line), HEK-293T cells (human embryonic kidney cell line) and BHK-21 cells (baby hamster kidney cell line) were cultured and maintained in Dulbecco’s modified Eagle medium (DMEM; Sigma) supplement with 10% fetal bovine serum (FBS; Gibco), 100 U/mL penicillin, and 100 μg/mL streptomycin in a 5% (vol/vol) CO_2_ incubator at 37 °C. The different knockdown BV2 cell lines were engineered by the CRISPR–Cas9 system. Knockdown cells were grown under conditions similar to that of naïve cells but appended with 2 μg/mL puromycin (InvivoGen). The JEV P3 strain used in this study was propagated in our laboratory. The titer of virus was determined by plaque assay on BHK-21 cells.

Primary microglial cells were isolated from BALB/c mice pups of either sex (postnatal days 0–1), as previously reported [30]. The whole brain cortex was dissected from the mouse brain, and the meninges were removed under a dissecting microscope. Tissue was digested using trypsin-DNase solution with a brief mechanical dissociation to obtain a cell suspension. The cell suspension was passed through 100-mm cell strainers and centrifuged at 400 × g for 8 min to obtain a cell pellet. The mixed cells were seeded in 75 T flasks and grown in low or high glucose DMEM (1000 mg/L glucose or 4500 mg/L glucose, respectively) supplemented with 10% FBS, 100 unit/mL penicillin, and 100 μg/mL streptomycin. Mixed glial cultures were maintained in a 5% CO_2_ incubator at 37 °C for 3–4 weeks. Mixed glial cultures were incubated with 0.25% trypsin diluted 1:4 in serum-free DMEM for 20–30 min in a 5% CO_2_ incubator at 37 °C. The upper layer of astrocytes was discarded, and the remaining microglial were used for analysis.

### JEV infection

Adult 6-week-old BALB/c mice were purchased from the Laboratory Animal Center of Huazhong Agricultural University, Wuhan, China. Mice were randomly divided into two groups (*n* = 9 /group): JEV-infected group and control group. Mice belonging to the JEV-infected group were intracerebrally injected with 200 plaque-forming units (PFU) of JEV P3 strain in 20 μL DMEM, whereas mice assigned to control group were injected intracerebrally with an equal amount of DMEM. At 0, 1, 3 and 5 dpi, mice from both groups were anesthetized with 2% isoflurane, followed by cervical dislocation, and the brain tissues were collected for further experiments. BV2 cells were plated in well plates, when cells grown to 90% confluence, the cell medium was removed and cells were infected with medium or JEV at an MOI of 5 for 90 min. Primary microglial cells plated on culture plates were treated similarly with BV2 cells.

### Plaque assay

Virus titers in cell culture supernatants were assessed by plaque assay in BHK-21 cells. The supernatants of cell cultures were harvested and stored at − 80 °C. Samples were serially diluted by serum-free DMEM and inoculated on the monolayers of BHK-21 cells at 37 °C for 1 h. The supernatants were removed and cells were washed thrice with serum-free DMEM, then the infected cells were further incubated for 4 days in DMEM containing 3% FBS and 1.5% sodium carboxymethyl cellulose (Sigma). Finally, viral titers were calculated based on visible plaques after staining with crystal violet. All data are expressed as the mean of triplicate samples.

### Antibodies and inhibitors

Mouse monoclonal antibodies against JEV-NS5 protein, monoclonal antibodies against JEV E protein and FITC-labeled JEV-E antibodies were generated in our laboratory. Commercially obtained antibodies used were rabbit monoclonal anti-EZH2 (Cell Signaling Technology), rabbit monoclonal anti-H2K27me3 (ABclonal Technology), rabbit polyclonal anti-Histone H3 (ABclonal Technology), rabbit polyclonal anti-Rnf19a (ABclonal Technology), mouse monoclonal anti-Iba1 (Abacm), rabbit monoclonal anti-RIG-I (ABclonal Technology), mouse monoclonal anti-GAPDH (ABclonal Technology), rabbit monoclonal anti-ubiquitin (ABclonal Technology), rabbit monoclonal anti-HA-Tag (Proteintech), mouse monoclonal anti-DDDDK-Tag (ABclonal Technology), EZH2 inhibitor (GSK343, 10 μM, Selleck), lysosome inhibitor (NH_4_Cl, 10 mM, Sigma) and proteasome inhibitor (MG132, 10 μM, Sigma) in our experiments.

### Immunohistochemistry (IHC) and immunofluorescence assay

The brain tissues collected from each group were immobilized in formalin, and then embedded in paraffin for coronal sections. The sections were used for IHC and IF assay. For IHC assay, brain sections were incubated with JEV E monoclonal antibodies at 4 °C overnight, and slides were stained by AEC Peroxidase Substrate Kit followed by hematoxylin staining. For IF assay, brain sections were incubated both with mouse Iba1 monoclonal antibodies and rabbit H3K27me3 monoclonal antibodies at 4 °C overnight, the secondary antibodies were used Alexa Fluor 555 goat anti-rabbit IgG, Alexa Fluor 647 goat anti-mouse IgG and DAPI. After being washed, slides were incubated with FITC-labeled JEV-E monoclonal antibodies.

### Enzyme-linked immunosorbent assay (ELISA)

The cell culture supernatants were collected from treated cells, after centrifugation of 500 g for 5 min, supernatants were collected and stored at − 80 °C. The supernatant protein levels of inflammatory cytokines (TNF-α, CCL5 and IL-6) were determined by ELISA kit according to the manufacturer’s instructions. TNF-α, CCL5 and IL-6 ELISA kits were purchased from Proteintech.

### Immunoblot assay

Total cellular lysates were generated with RIPA buffer (Sigma) containing protease inhibitors (Roche). Protein concentrations were determined using a BCA protein assay kit (Thermo Scientific). Equal protein quantities were separated by SDS-PAGE and transferred to a polyvinylidene fluoride membrane (Millipore) using a Mini Trans-Blot Cell (Bio-Rad). Blots were probed with the relevant antibodies, and proteins were detected using enhanced chemiluminescence reagent (Thermo Scientific).

### RNA extraction and quantitative real-time PCR

Total RNA was extracted from treated cells with TRIzol reagent (Magen) according to the manufacturer’s instructions, and 1ug of RNA was used to synthesize cDNA using a First-Strand cDNA Synthesis Kit (ABclonal Technology). Quantitative real-time PCR was performed using a ViiA™ 7 Real-time PCR System (Applied Biosystems) and SYBR Green Real-time PCR Master Mix (ABclonal Technology). Amplification was performed for 2 min at 50 °C and 10 min at 95 °C, followed by 40 cycles of 95 °C for 15 s, 60 °C for 15 s, and 72 °C for 30 s. The relative expression levels of mRNA were normalized to that of β-actin within each sample using the 2 − ΔΔCt method. Primers used are listed in Table 1.

**Table 1:** Primer pairs used for quantitative real-time RT-PCR analyses

Primer name	Sequence (5′ → 3′)
β-actin-F	CACTGCCGCATCCTCTTCCTCCC
β-actin-R	CAATAGTGATGACCTGGCCGT
TNF-α-F	TGTCTCAGCCTCTTCTCATTCC
TNF-α-R	TTAGCCCACTTCTTTCCCTCAC
IL-6-F	CTGCTTCTGGTGATGGCTACTG
IL-6-R	GGCATCACCTTTGGCATCTT
CCL5-F	GCTGCTTTGCCTACCTCTCC
CCL5-R	TCGAGTGACAAACACGACTGC
IP-10-F	CCTGCTGGGTCTGAGTGGGA
IP-10-R	GATAGGCTCGCAGGGATGAT
MCP-1-F	GCTTCTGGGCCTGCTGTTCA
MCP-1-R	AGCTCTCCAGCCTACTCATT
JEV-C-F	ATGCTGAAACGCGGATTACC
JEV-C-R	GCGTCGATGAGTGTTCCAAG
Rnf19a-F	AACGCAGCCATAGAGAAGGG
Rnf19a-R	TCATCCACACTGCTGCTTCC

F for forward primer, R for reverse primer

### Construction of BV2 knockdown cell lines using CRISPR/Cas9 technology

The sequences of specific sgRNA for target genes were predicted by using sgRNAcas9 software (www.biootools.com). Two oligos for the sgRNA of EZH2 5′-GAATGCAGTCGCCTCGGTGC-3′ and 5′-GCACCGAGGCGACTGCATTC-3′ or Rnf19a 5′-TCCTGTGTACGTGGGCCGCA-3′ and 5′ -TGCGGCCCACGTACACAGGA-3′ were cloned into the lentiCRlSPRv2 plasmid (Addgene, #52961). The virus was packaged in HEK-293T cells. In 6-well plate, 140 μL opti-MEM I (invitrogen), 800 ng lentiCRlSPRv2 (Contains sgRNA), 800 ng psPAX2 (Addgene #12260) and 400 ng pMD2.G (Addgene #122591) mix well, and then add 8 μL Fugene HD transfection reagent (150 μL total). 48 h later, spun at 3000 rpm for 15 min to harvest the supernatant. BV2 cells were seeded in 6-well plate, and then BV2 cells were infected with lentivirus at 24 h with 8 μg/mL polybrene. At 24 h post-infection, puromycin (3.5 μg/mL) was added for selection. Knockdown Cell lines were confirmed by immunoblot assay.

### Chromatin immunoprecipitation-sequencing (ChIP) assay

ChIP assays followed the reported protocol by Giaimo and Huang. In brief, BV2 cells in 10-cm tissue culture dishes were infected with JEV. At 24 hpi, cells were harvested and cross-linked with 1% formaldehyde for 15 min at room temperature. The cross-linking reaction was stopped by addition of 125 mM glycine (final concentration) for 5 min, and cells were chilled on ice. Then pellet cells by centrifugation for 5 min at 4 °C and ~ 271 × g and wash with 1 × phosphate-buffered saline (PBS). The cell pellet was resuspended in 1 × SDS lysis buffer (1% SDS, 10 mM EDTA, 50 nM Tris–HCl, pH 8.1, and protease inhibitor mixture) and incubated on ice for 10 min. The lysates were sonicated for 2 min to fragment DNA to an average length of 400–800 bp, and subsequently diluted to tenfold with 1 × ChIP buffer (0.01% SDS, 1% Triton X-100, 1.2 mM EDTA, 16.7 mM Tris–HCl [pH 8.1], 150 nM NaCl and protease inhibitor mixture). Cellular debris was spun down, and the supernatant was diluted tenfold with 1 × ChIP dilution buffer (0.01% SDS, 1% Triton X-100, 1.2 mM EDTA, 16.7 mM Tris–HCl, pH 8.1, 150 nM NaCl, and protease inhibitor mixture) and incubated with 5 μg of antibodies against H3K27me3 or control rabbit IgG antibody, with rotation overnight at 4 °C. Protein A/G beads (Invitrogen) were pre-blocked with 0.5 mg/mL BSA and 0.125 mg/mL calf thymus DNA for 1 h at 4 °C, and then added to the lysate–antibody mixture for another incubation for 2 h at 4 °C. The protein A-Sepharose beads were recovered by centrifugation (1280 × g for 4 min) and washed with 1 mL of each of the following wash buffers in the following order: (i) once with low-salt immune complex wash buffer (0.1% SDS, 1% Triton X-100, 2 mM EDTA, 20 mM Tris–HCl, pH 8.1, 150 mM NaCl); (ii) once with high-salt immune complex wash buffer (0.1% SDS, 1% Triton X-100, 2 mM EDTA, 20 mM Tris–HCl, pH 8.1, 500 mM NaCl); (iii) once with LiCl buffer (0.25 M LiCl, 1% NP-40 alternative, 1% Na-deoxycholate, 1 mM EDTA, 10 mM Tris–HCl, pH 8.1); and (iv) twice with TE buffer (10 mM Tris–HCl, pH 8.1, 0.1 mM EDTA). Immunoprecipitated protein–DNA complexes were eluted twice with fresh elution buffer (1% SDS, 0.1 m NaHCO_3_) for 1 h and 15 min, respectively, at room temperature. To reverse the cross-linking, the eluted samples were incubated at 65 °C for overnight in the presence of 0.2 M NaCl. The eluted samples were then treated with proteinase K, and the DNA species were precipitated by using phenol–chloroform. The final DNA pellet was dissolved in TE buffer and quantified by qPCR. Input (1%) was used for qPCR analysis. The results were normalized to input values (percent input = 2 [(Ct, Input − Ct, IP) × 100]). The relative enrichment levels indicate the fold changes over the IgG control. The following primers were used in the ChIP assays: Rnf19a forward, GCTGCCCTCAGACTGTCATAC; Rnf19a reverse, TGTCTCCCTTCTGTTGGGGT; Cd2ap forward, CCCTGGGCCAAGCATATTCA; Cd2ap reverse, AGGATCCTGTTGCAGTGTCG; Ipo11 forward, GCATCTCTGAAACGTTATC; Ipo11 reverse, CACTGCAAAGGCTGCCCACC.

### Chromatin immunoprecipitation-sequencing (ChIP-seq) and data analysis

Libraries for ChIP-seq were generated using the MGIeasy kit (BGI, 100006986) according to the instructions of the manufacturer’s protocol and the paired-end 150-bp sequencing was conducted by MGISEQ-2000RS (BGI). The low-quality reads and adaptor sequences were removed by Trimmomatic (v0.39) software. Then the clean reads were mapped to the mouse mm10 genome by bowtie2 (2.3.4) with the default parameters. Samtools (v1.7) was used to filter out the unmatched reads and the read duplicates were removed by Picard tools (v2.18) was used to remove the duplicated reads. MACS2 (v2.2) was utilized to call peaks and the differential peaks were found by the R packages DiffBind (v3.0.9). Differential ChIP-seq peaks were selected based on the following criteria: |log2 fold change|≥ 1 and FDR ≤ 0.05. The heatmap and volcano plots were generated using the R package pheatmap (v1.0.12) and ggplot2 (v3.2.1) for visualizing the different genes. The peaks were annotated by ChIPseeker (v1.26) for gene category analysis and Clusterprofiler (v3.12.0) for gene function annotation such as KEGG and GO analysis.

### Cell viability assay

The viability of cultured cells was detected using the Cell Titer-Glo One Solution Assay kit (Promega) according to the manufacturer’s instruction. The quantification of luminescence signals was calculated based on the reported method [31].

### Co-immunoprecipitation and immunoblot assays

HEK-293T cells were transfected with the indicated combinations of plasmids and collected 48 h after transfection. Cells were lysed for 30 min with 500 μL lysis buffer containing protease inhibitor. A part of cell lysates (40 μL) were subject to immunoblot assay to detect the expression of target proteins, and the rest of cell lysates were incubated with IgG and protein A + G agarose to remove nonspecific reaction. The supernatant after centrifugation was incubated with DDDDK-Tag antibody and protein A + G agarose overnight at 4 °C. The unbound proteins and antibodies in agarose beads were washed by PBS five times and eluted with 2 × SDS loading buffer by boiling for 5 min. Proteins isolated from the beads and the cell lysates were subjected to immunoblot assay.

### Ubiquitination assays

To analyze the effect of Rnf19a on the ubiquitination of RIG-I in transfected cells, HEK-293T cells were transfected with pCAGGS-Rnf19a, pCAGGS-Flag-RIG-I and HA-tagged ubiquitin. The proteasome inhibitor MG132 was added 24 h after transfection for 4 h, and the remaining steps are roughly the same as the co-immunoprecipitation steps described above.

### The luciferase assay

HEK-293T cells were seeded in a 48-well plate and transfected with the indicated plasmid and a luciferase reporter plasmid luc-NF-κB together with pRL-TK (Renilla luciferase plasmid) as a control reporter vector. At 48 h post-transfection, the cells were disrupted with lysis buffer (Promega), and luciferase activity was measured by the Dual-Luciferase Reporter Assay (Promega) on a Synergy 2 microplate reader (BioTek). The results were calculated by normalization of firefly luciferase activity to Renilla luciferase activity.

### Statistical analysis

All results are expressed as mean ± SEM. Statistical analyses were performed by using the GraphPad Prism 6 software. Statistical significance was determined with a two-sided unpaired *t*-test for two groups.

## Results

### JEV infection increases H3K27me3 modification in microglial cells

**Fig. 1:**
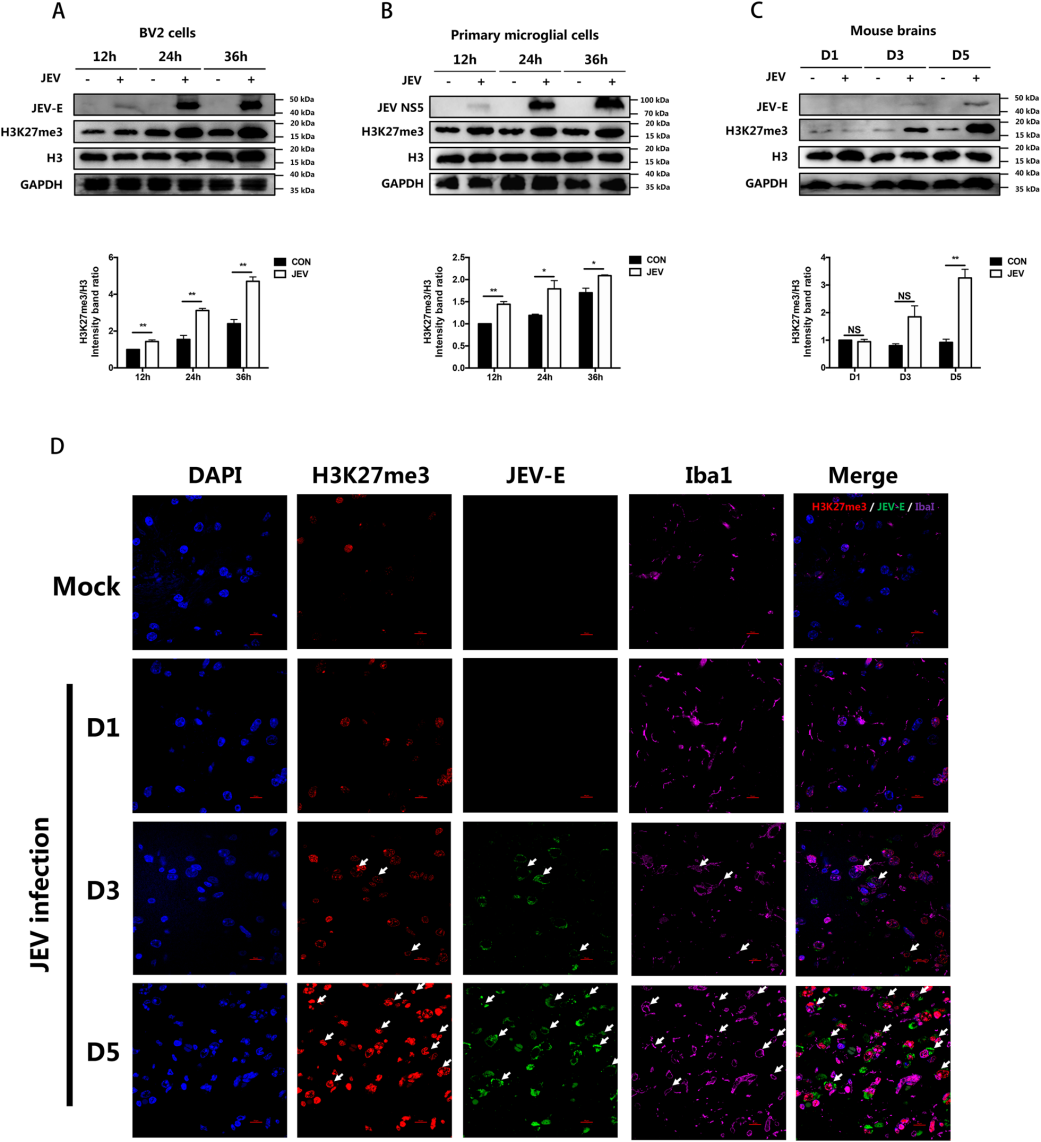
JEV infection elevates H3K27me3 modification in microglial cells. **A, B** BV2 cells (**A**) and primary mouse microglial cells (**B**) were infected with JEV at an MOI of 5. At 12, 24 and 36 hpi, cells were lysed for protein extraction, and the H3K27me3 modification was detected by immunoblotting (upper panel). Levels of H3K27me3 were quantified by immunoblotting scanning using Image J software and normalized to the amount of histone H3 (lower panel). **C, D** 6-week-old BALB/c mice (*n* = 9 /group) were intracerebrally injected with 200 PFU of JEV P3 strain in 20 μL DMEM or equal amount of DMEM (mock infected). The mouse brains were collected at day 1, 3 and 5 post-infection. **C** H3K27me3 levels and the expression of JEV E protein were detected by immunoblotting (upper panel). H3K27me3 levels in different mice brain samples were quantified by immunoblotting scanning using Image J software and normalized to the amount of histone H3 (lower panel). **D** Coronal sections of brain tissues were subjected to immunofluorescence assay by using antibodies against microglia marker Iba1 (purple), JEV E protein (green) and H3K27me3 (red). Scale bar, 10 μm. Data are expressed as means ± SEM from three independent experiments. *p < 0.05, **p < 0.01, NS represents no significant difference

To study the effect of JEV infection on H3K27me3 modification in microglia, BV2 mouse microglial cell line and primary mouse microglial cells were infected with JEV at MOI of 5. The productive infection of JEV in both cells was determined by plaque assay (Additional file 1: Fig. S1A and S1B). Immunoblotting assay was then used to detect H3K27me3 modification. Upon JEV infection, a notable time-dependent increase in H3K27me3 level was observed in both BV2 and primary mouse microglial cells (Fig. 1A and B).

To further verify this finding in vivo, 6-week-old BALB/c mice were injected with JEV intracerebrally. The mouse brains were collected at 1, 3 and 5 days post-infection (dpi) and subjected to plaque assay and immunoblotting assay. The results showed that JEV efficiently replicated in the mouse brain (Additional file 1: Fig. S1C). Moreover, the level of H3K27me3 in brain tissues of JEV-infected mice was shown to increase at both 3 and 5 dpi, while no significant change was observed at 1 dpi (Fig. 1C). To determine whether microglia were responsible for the increased H3K27me3 in JEV-infected mouse brain, brain sections were immunostained using anti-JEV E antibody. The results showed that JEV E protein was detected in both the cortex and thalamus regions on day 3 post-infection, with a higher amount of viral protein detected in these regions and a small amount in the hippocampus region on day 5 dpi (Additional file 2: Fig. S2). Then brain sections including cortex and hypothalamus regions were utilized for an immunofluorescence assay to detect the expression of H3K27me3 in JEV-infected microglia. The results showed that there was a noticeable increase in the level of H3K27me3 in JEV E protein positive microglia when compared to uninfected cells (Fig. 1D). Collectively, these results suggest that JEV infection leads to an upregulation of H3K27me3 modification in mouse microglia.

### H3K27me3 positively regulates the inflammatory response in microglial cells during JEV infection

**Fig. 2:**
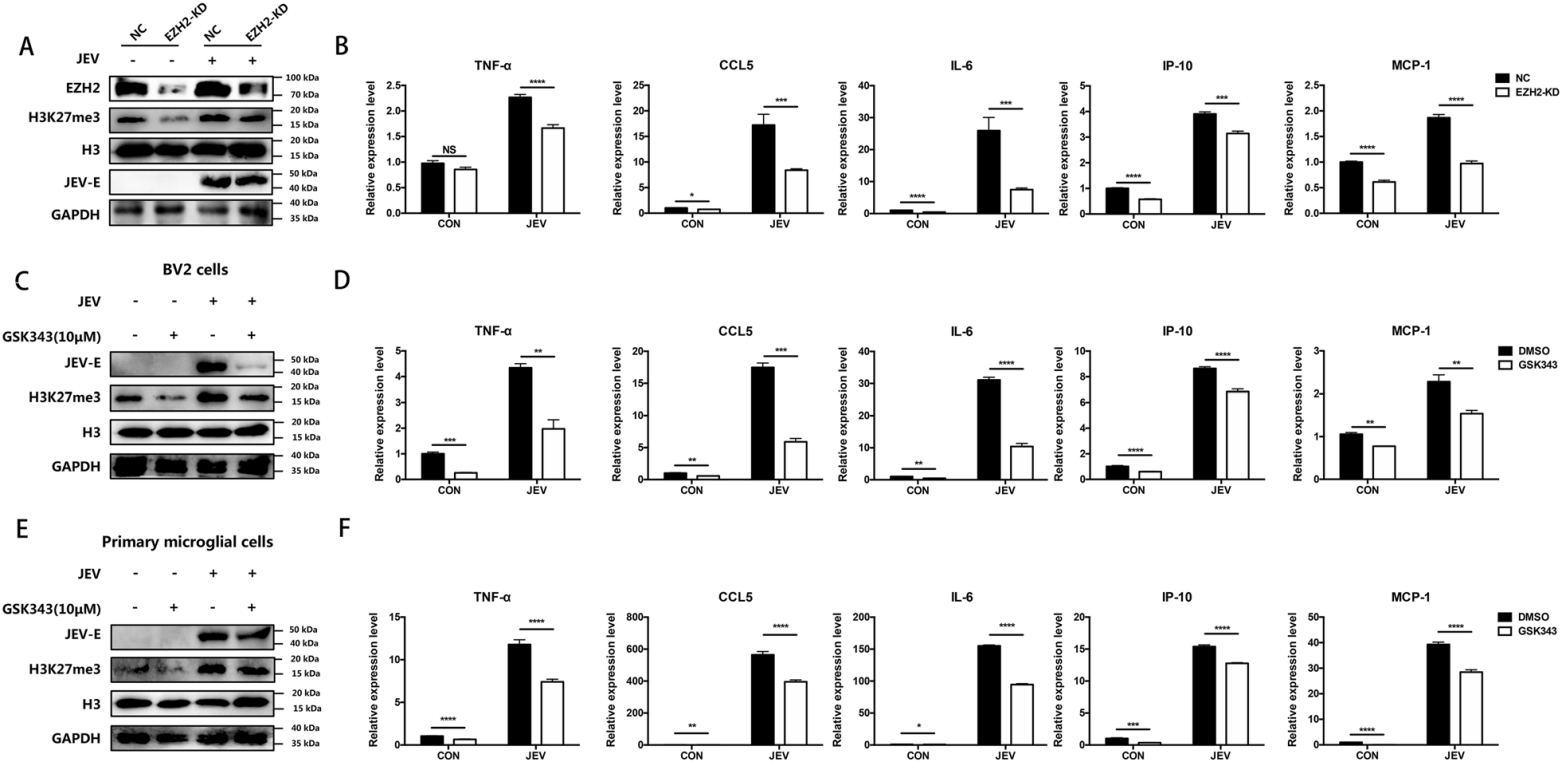
H3K27me3 promotes JEV-induced inflammatory response in microglial cells. **A, B** EZH2 knockdown (EZH2 KD) BV2 cell line or negative control (NC) cells were infected with JEV at an MOI of 5, and cells were harvested at 36 hpi. The protein expression of JEV E, EZH2 and H3K27me3 was detected by immunoblotting (**A**). RNA was extracted and analyzed by qRT-PCR to measure mRNA levels of inflammatory cytokines (**B**). **C, D** BV2 cells were treated with GSK343 (10 μM) or equal volume of DMSO following JEV infection, and cells were harvested at 36 hpi. The levels of H3K27me3 were measured by immunoblotting (**C**) and mRNA abundance of inflammatory cytokines was examined by qRT-PCR (**D**). **E, F** Primary microglial cells were treated with GSK343 (10 μM) or equal volume of DMSO following JEV infection, and cells were harvested at 36 hpi. The levels of H3K27me3 were measured by immunoblotting (**E**) and mRNA abundance of inflammatory cytokines was determined by qRT-PCR (**F**). Data are expressed as means ± SEM from three independent experiments. *p < 0.05, **p < 0.01, ***p < 0.001, ****p < 0.0001, NS represents no significant difference

Given that the histone methyltransferase EZH2 is specifically responsible for H3K27me3 catalyzation [32], EZH2 knockdown (EZH2 KD) BV2 cell line was generated by CRISPR/Cas9 approach to investigated the role of H3K27me3 on JEV-induced inflammation in microglia. After confirming that EZH2 knockdown does not affect the cell viability (Additional file 3: Fig. S3), EZH2 KD and negative control (NC) cells were infected with JEV at an MOI of 5. The immunoblotting results showed that the H3K27me3 modification was downregulated after EZH2 knockdown in both JEV-infected and uninfected cells (Fig. 2A). Then, the expression of pro-inflammatory cytokines in EZH2 KD and NC cells after JEV infection was examined. The result showed a significant reduction of TNF-α, CCL5, IL-6, IP-10 and MCP-1 mRNA levels induced by JEV infection in EZH2 KD cells (Fig. 2B). Consistently, the protein production of TNF-α, CCL5 and IL-6 in supernatant of EZH2 KD cells was also decreased (Additional file 4: Fig. S4A). These results suggesting H3K27me3 modification contributes to JEV-induced inflammation in microglia. Then the effect of H3K27me3 modification on JEV replication was determined by plaque assay. No obvious difference in viral replication was observed in EZH2 KD and NC cells (Additional file 5: Fig. S5A), suggesting that the effect of H3K27me3 modification on inflammation was not caused by affecting JEV replication.

To further verify our results, BV2 cells were treated with nontoxic dose of EZH2 inhibitor GSK343 (10 μM) (Additional file 6: Fig. S6A) following JEV infection, and the expression of pro-inflammatory cytokines was measured by qRT-PCR at 36 hpi. As expected, treatment of EZH2 inhibitor reduced H3K27me3 level (Fig. 2C) as well as mRNA levels of TNF-α, CCL5, IL-6, IP-10 and MCP-1 (Fig. 2D) in JEV-infected cells. Similarly, treatment of primary microglial cells with nontoxic dose of GSK343 (10 μM) (Additional file 6: Fig. S6B) also led to significantly decrease of H3K27me3 level (Fig. 2E) and mRNA abundance of pro-inflammatory cytokines (Fig. 2F) during JEV infection. In consistent with the result shown in EZH2 KD cells, GSK343 treatment also did not affect JEV replication (Additional file 5: Fig. S5B). Taken together, our data demonstrate that EZH2-mediated H3K27me3 plays a positively regulatory role in the neuroinflammatory response caused by JEV.

### Genome-wide ChIP-sequencing analysis of H3K27me3 target gene profile in JEV-infected microglial

As H3K27me3 is linked with transcriptional repression, it is possible that the elevated level of H3K27me3 may contribute to JEV-induced neuroinflammation by causing changes in gene transcription. Therefore, ChIP-sequencing was conducted to identify the genes with varying levels of H3K27me3 modifications during JEV infection. BV2 cells were mock-infected or infected with JEV at an MOI of 5, and then the cells were lysed and subjected to ChIP assay using anti-H3K27m3 or IgG antibody, followed by deep sequencing on the purified ChIP-DNA fragments.

**Fig. 3:**
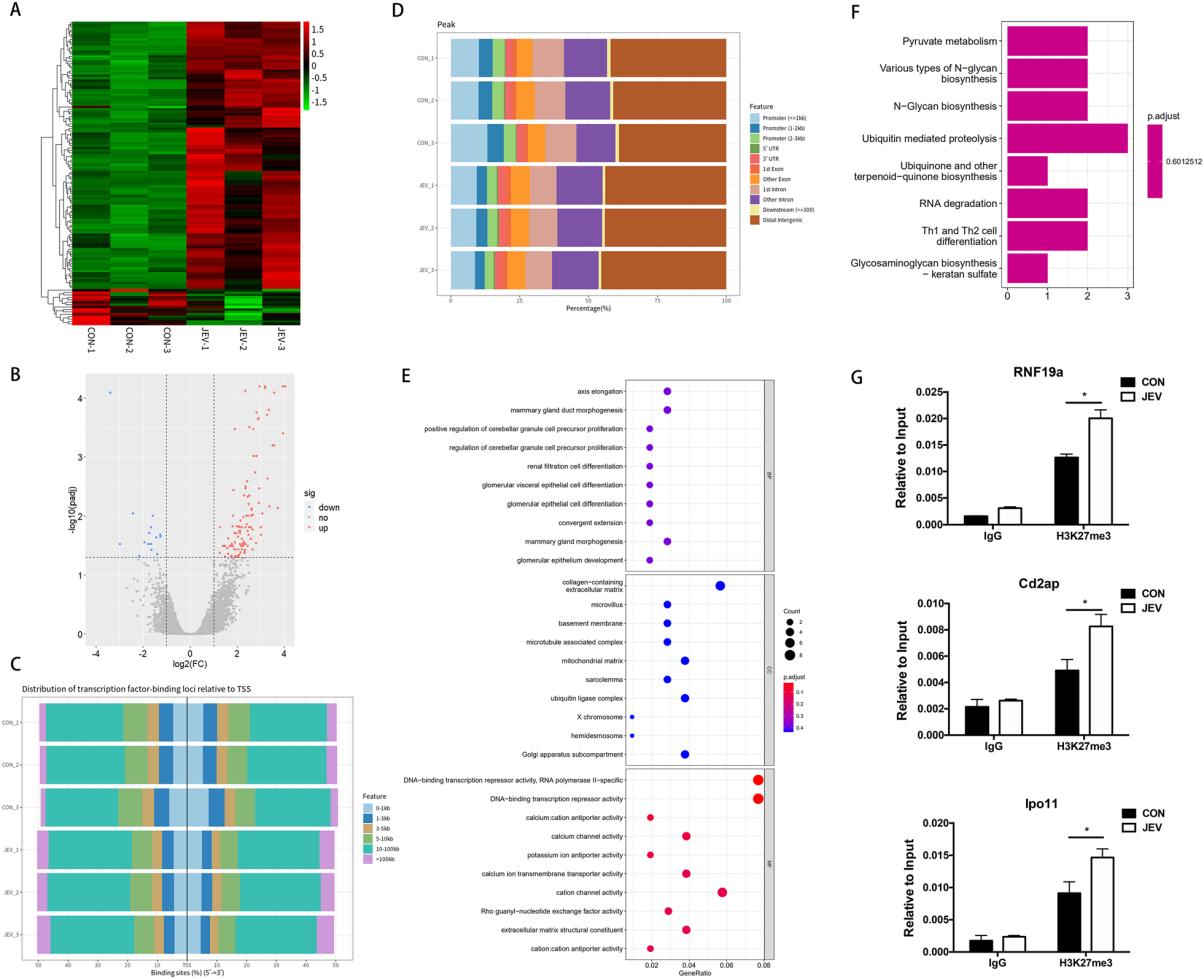
ChIP-sequencing analysis of the H3K27me3 target gene profile in BV2 cells infected with JEV and validation of ChIP-sequencing results. **A** The hierarchical clustering of differently expressed genes. The red color indicates upregulated genes and the green color indicates downregulated genes. **B** The volcano plot represents the modified genes. The grey dots denote non-significantly expressed genes. The upregulated genes are presented as red dots and the downregulated genes are presented as blue dots. **C** Distribution of peak binding sites to the TSS of the nearest gene. **D** The position of the genome annotation peak by barplot in terms of genome features. **E** GO term enrichment analysis for the differentially regulated genes. **F** KEGG pathway analysis for the differentially regulated genes. **G** The recruitment of H3K27me3 to the binding region of Rnf19a, Ipo11 and Cd2ap in BV2 cells. The binding region of Rnf19a, Ipo11 and Cd2ap were ChIP-ed with anti-H3K27me3 antibodies or IgG control. Data are expressed as means ± SEM from three independent experiments. *p < 0.05

According to the output files of the H3K27me3 dataset generated by MACS2 (see Additional file 7: S7), we obtained 126 significantly differentially regulated (fold change ≥ 2 and FDR ≤ 0.05) peaks in three JEV-infected cells compared to those in control cells (Table 2). Among the 126 significant peaks, 111 peaks showed an upregulated expression pattern, while 16 peaks were downregulated (Table 2). For generating the profile of ChIP peaks binding to transcriptional starting site (TSS) regions, the Heatmap of ChIP binding to TSS regions (Fig. 3A) and the volcano plot (Fig. 3B) were made to illustrate the global regulatory pattern of peaks upon JEV infection by using the R package. Then, the distance from the peak (binding site) to the TSS of the nearest gene was calculated and the distributions were visualized by ChIPseeker (Fig. 3C). Furthermore, to annotate the location of ChIP-peaks in terms of genomic features, the bar plots were provided to visualize the genomic annotation in different experimental groups (Fig. 3D). To gain insight into the biological function of differentially regulated genes, GO analysis and pathway enrichment analysis were carried out. Interestingly, the differentially regulated genes were mainly enriched in DNA transcriptional suppression in the molecular function classification category (Fig. 3E) and were involved in key pathways such as ubiquitination modification, RNA degradation and cell differentiation (Fig. 3F), indicating that H3K27me3 may promote JEV-induced inflammation via regulating ubiquitination or cell differentiation pathway.

**Table 2:** The different regulated genes from ChIP-sequencing result

Differences	Peak number	Proportion %
Upregulated	111	88
Downregulated	15	12
Total	126	100

To verify the ChIP-sequencing result, the top three genes Rnf19a, Ipo11 and Cd2ap in the enrichment list were selected to analyze their H3K27me3 levels at the binding regions by using ChIP assay coupled with qRT-PCR. As expected, the H3K27me3 levels on the binding regions of Rnf19a, Cd2ap and Ipo11 were significantly increased upon JEV infection in microglial cells (Fig. 3G), which confirms the reliability of ChIP-sequencing results.

### H3K27me3 modification promotes the inflammatory response induced by JEV via inhibiting Rnf19a expression

**Fig. 4:**
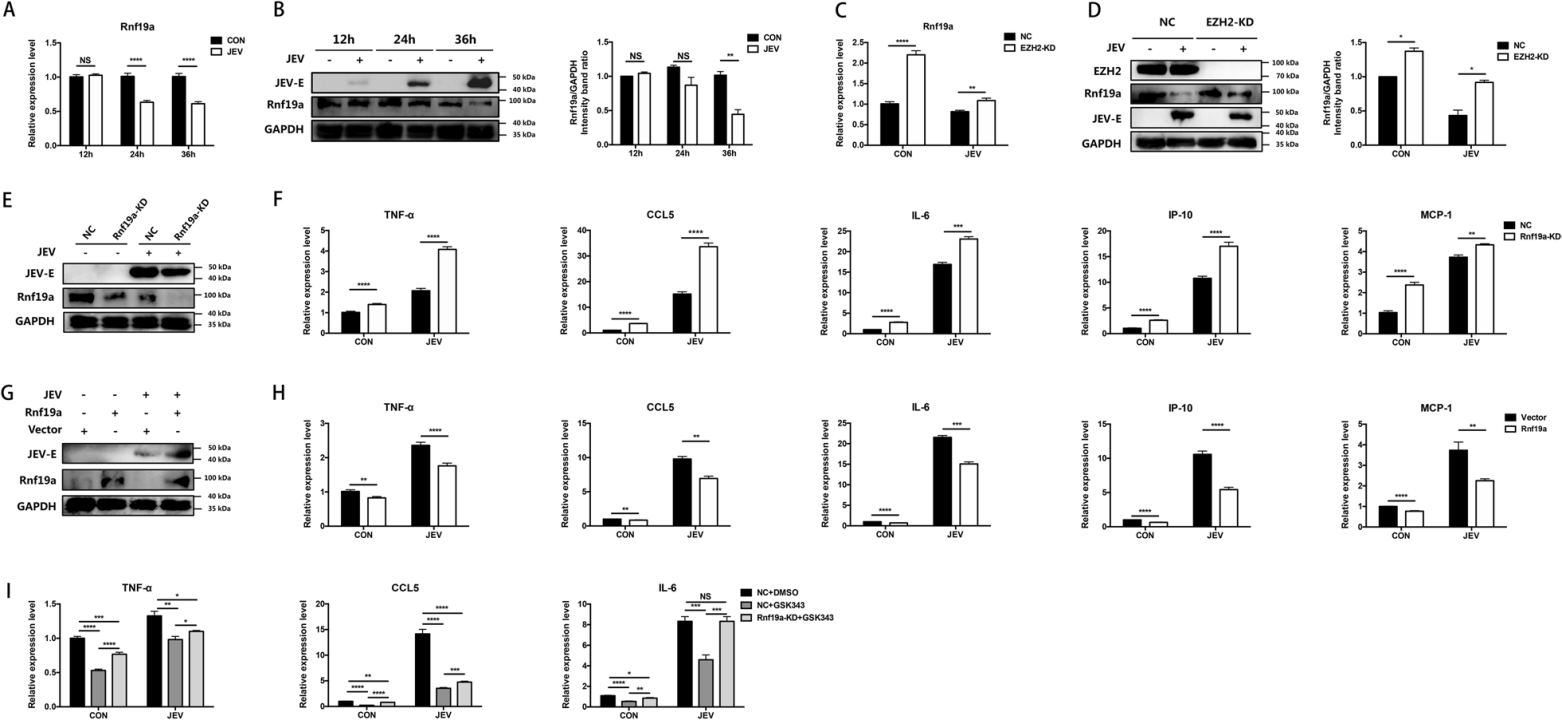
H3K27me3 modification of Rnf19a contributes to the JEV-induced inflammatory response in BV2 cells. **A, B** BV2 cells were infected with JEV at an MOI of 5, mRNA (**A**) and protein (**B**) levels of Rnf19a were measured at 12, 24 and 36 hpi. The protein level of Rnf19a was quantified by immunoblotting scanning using Image J software and normalized to the amount of GAPDH (right panel). **C, D** EZH2 knockdown (EZH2 KD) BV2 cells or negative control (NC) cells were infected with JEV at 5 MOI and cells were harvested at 36 hpi. RNA was extracted and analyzed by qRT-PCR to measure mRNA level of Rnf19a (**C**). The expression of Rnf19a was detected by immunoblotting (**D**), and quantified by immunoblotting scanning using Image J software and normalized to the amount of GAPDH (right panel). **E, F** Rnf19a knockdown (Rnf19a KD) BV2 cells or negative control (NC) cells were infected with JEV at an MOI of 5, and cells were harvested at 36 hpi. The expression of Rnf19a was detected by immunoblotting (**E**) and mRNA levels of inflammatory cytokines were examined by qRT-PCR (**F**). **G, H** BV2 cells were transfected with pCAGGS-Rnf19a plasmid or pCAGGS empty vector following JEV infection (MOI = 5). Cells were harvested at 36 hpi, and the expression of Rnf19a (**G**) and mRNA abundance inflammatory cytokines (**H**) were detected by immunoblotting and qRT-PCR, respectively. **I** Rnf19a knockdown (Rnf19a KD) or negative control (NC) BV2 cells were treated with GSK343 (10 μM) or equal volume of DMSO following JEV infection at MOI of 5. At 36 hpi, the mRNA abundance of inflammatory cytokines was determined by qRT-PCR. Data are expressed as means ± SEM from three independent experiments. *p < 0.05, **p < 0.01, ***p < 0.001, ****p < 0.0001, NS represents no significant difference

Given that Rnf19a, a member of the E3 ubiquitin ligase family, is the most enriched gene, it is worth investigating its potential role in regulating the JEV-induced neuroinflammation. To this end, mRNA and protein abundance of Rnf19a were determined by qRT-PCR and immunoblot assay. The results showed that mRNA level of Rnf19a began to decrease at 24 hpi (Fig. 4A), and protein level was significantly decreased at 36 hpi in JEV-infected BV2 cells (Fig. 4B). To investigate whether the expression of Rnf19a was regulated by EZH2-mediated H3K27me3, EZH2 knockdown BV2 cells were infected with JEV at an MOI of 5, and then the mRNA and protein levels of Rnf19a were measured. We found that knockdown of EZH2 elevated both transcriptional and translational levels of Rnf19a at 36 hpi (Fig. 4C and D), suggesting that EZH2-mediated H3K27me3 contributes to transcriptional repression of Rnf19a during JEV infection.

To clarify the role of Rnf19a in JEV-induced neuroinflammation, Rnf19a knockdown (Rnf19a KD) BV2 cell line was generated by using CRISPR–Cas9 approach (Fig. 4E). It was shown that Rnf19a knockdown does not affect the viability of BV2 cells (Additional file 3: Fig. S3). Subsequently, production of pro-inflammatory cytokines in JEV-infected Rnf19a KD and NC BV2 cells was measured by qRT-PCR and ELISA. The results showed that knockdown of Rnf19a increased the transcription of TNF-α, CCL5, IL-6, IP-10 and MCP-1 (Fig. 4F) in response to JEV infection. Consistently, the concentrations of TNF-α, CCL5 and IL-6 in cell supernatant also elevate after JEV infection (Additional file 4: Fig. S4B). Plaque assay suggests that Rnf19a knockdown does not influence the replication of JEV in BV2 cells (Additional file 5: Fig. S5C). To substantiate this finding, Rnf19a was overexpressed in BV2 cells (Fig. 4G) following by JEV infection. Consistently, overexpression of Rnf19a could significantly reduce the expression of TNF-α, CCL5, IL-6, IP-10 and MCP-1 during JEV infection (Fig. 4H). Collectively, these results suggest that Rnf19a plays a negative regulatory role in inflammatory response induced by JEV.

To explore whether H3K27me3 modification promotes JEV-induced inflammatory response via inhibiting Rnf19a expression, Rnf19a KD and NC BV2 cells were treated with EZH2 inhibitor, following JEV infection, and the mRNA abundance of pro-inflammatory cytokines was determined by qRT-PCR. An inhibition of the downregulation of inflammatory cytokines caused by GSK343 treatment after JEV infection was observed in Rnf19a KD cells (Fig. 4I), suggesting that Rnf19a plays a role in the regulation of H3K27me3 modification on JEV-induced inflammatory response.

### Rnf19a modulates the inflammatory response induced by JEV through mediating ubiquitination and degradation of RIG-I

**Fig. 5:**
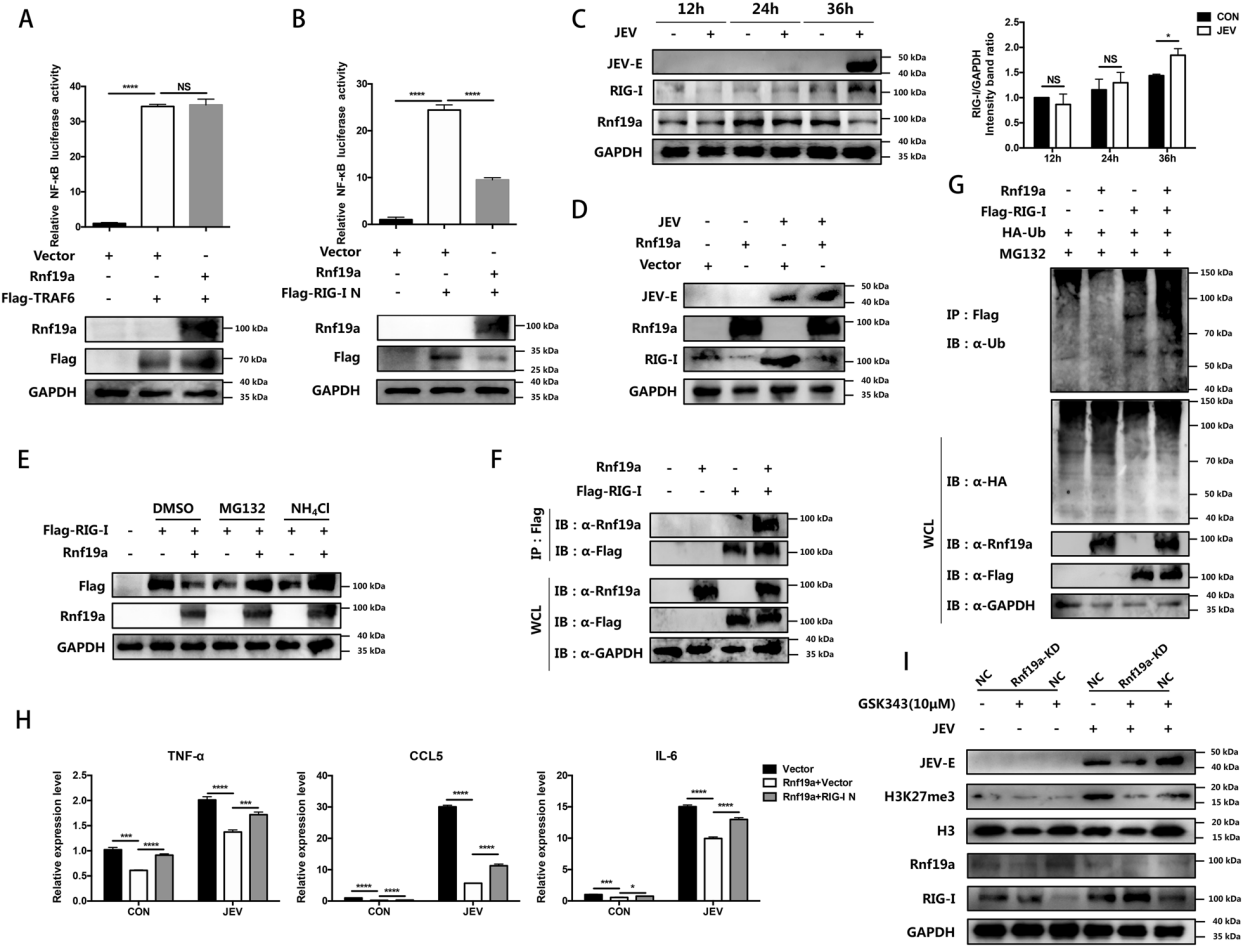
Rnf19a regulates JEV-induced inflammatory response in BV2 cells by mediating RIG-I degradation. **A** HEK-293T cells were co-transfected with luc-NF-κB reporter, pRL-TK construct together with pCAGGS-Flag-TRAF6 plasmid, pCAGGS-Rnf19a plasmid or pCAGGS empty vector. The luciferase activity was determined, and the expression of indicated proteins was detected by immunoblot assay at 36 h post-transfection. **B** HEK-293T cells were co-transfected with the luc-NF-κB reporter, pRL-TK construct and pCAGGS-Flag-RIG-I N plasmid, pCAGGS-Rnf19a plasmid or pCAGGS empty vector. The luciferase activity was determined and the expression of indicated proteins was detected by immunoblot assay at 36 h post-transfection. **C** BV2 cells were infected with JEV at an MOI of 5. At 12, 24 and 36 hpi, the expression of RIG-I and Rnf19a were determined by immunoblotting (left panel), and quantified by immunoblotting scanning using Image J software and normalized to the amount of GAPDH (right panel). **D** BV2 cells were transfected with pCAGGS-Rnf19a plasmid or pCAGGS empty vector. The protein level of RIG-I was determined by immunoblot assay at 36 h post-transfection. **E** HEK-293T cells were co-transfected with pCAGGS-Flag-RIG-I plasmid and pCAGGS-Rnf19a plasmid or pCAGGS empty vector. At 24 h post-transfection, cells were treated with DMSO, the proteasome inhibitor MG132 (10 μM) or the lysosome inhibitor NH_4_Cl (10 mM). After incubation for 4 h, the protein levels of RIG-I and Rnf19a were determined by immunoblot assay. **F** HEK-293T cells were transfected with pCAGGS-Flag-RIG-I plasmid along with pCAGGS empty vector or pCAGGS-Rnf19a. The cell lysates were subjected to denaturing immunoprecipitation with anti-Flag beads and the products were analyzed by immunoblotting with the indicated antibodies. **G** HEK-293T cells were co-transfected with pCAGGS-Flag-RIG-I plasmid, pCAGGS empty vector or a pCAGGS-Rnf19a construct and plasmid encoding HA-WT-ubiquitin (Ub). Followed by treatment with MG132, cells lysates were subjected to denaturing immunoprecipitation with anti-Flag beads and products were analyzed by immunoblotting with indicated antibodies. **H** BV2 cells were co-transfected with pCAGGS-Rnf19a plasmid and pCAGGS-Flag-RIG-I N plasmid or pCAGGS empty vector following JEV infection at an MOI of 5. At 36 hpi, the mRNA abundance of inflammatory cytokines was measured by qRT-PCR. **I** Rnf19a knockdown (Rnf19a KD) or negative control (NC) BV2 cells were treated with GSK343 (10 μM) or equal volume of DMSO following JEV infection at MOI of 5. Data are expressed as means ± SEM from three independent experiments. *p < 0.05, ***p < 0.001, ****p < 0.0001, NS represents no significant difference

In previous studies, it was found that NLRP11 recruits the ubiquitin ligase Rnf19a to catalyze K48-linked ubiquitination of TRAF6. This leads to the degradation of TRAF6, which in turn negatively regulates NF-κB signaling [33]. To further clarify how Rnf19a regulates inflammatory response during JEV infection, HEK-293T cells were co-transfected with a NF-κB luciferase reporter vector, Rnf19a construct, and plasmid encoding either TRAF6 or the N-terminal CARDs of RIG-I. Surprisingly, we found that Rnf19a interfered with RIG-I N instead of TRAF6 induced activation of NF-κB (Fig. 5A and B), indicating that Rnf19a may modulate the inflammatory response by inhibiting RIG-I signaling during JEV infection. Then we examined the dynamic changes of Rnf19a and RIG-I expression in BV2 cells during JEV infection by immunoblot assay. As expected, RIG-I was upregulated when Rnf19a was downregulated after JEV infection (Fig. 5C). To explore whether RIG-I expression could be regulated by Rnf19a, BV2 cells were transfected with Rnf19a construct, and the expression of RIG-I was examined. The result showed that overexpression of Rnf19a reduced the expression of endogenous RIG-I (Fig. 5D). Considering that Rnf19a is an E3 ubiquitin ligase, we proceeded to investigate whether the decrease in RIG-I protein level was due to degradation facilitated by Rnf19a through the ubiquitin proteasome pathway or lysosome pathway. We found that treatment with proteasome inhibitor MG132 and lysosome inhibitor NH_4_Cl both inhibited RIG-I reduction induced by Rnf19a overexpression (Fig. 5E), suggesting that Rnf19a-mediated degradation of RIG-I is dependent on both proteasome and lysosome pathways. Then a possible interaction between Rnf19a and RIG-I was analyzed by Co-IP assay. HEK-293T cells were co-transfected with plasmid encoding Flag-tagged RIG-I and Rnf19a construct, and the cell lysates were immunoprecipitated with anti-Flag antibody and analyzed by immunoblotting using anti-Flag antibody and anti-Rnf19a antibody. The result revealed an interaction between Rnf19a and RIG-I (Fig. 5F). To further verify the Rnf19a-mediated ubiquitination of RIG-I, HEK-293T cells were co-transfected with Flag-RIG-I, Rnf19a and HA-ubiquitin plasmids. The immunoprecipitation result showed that Rnf19a facilitates the ubiquitination of RIG-I (Fig. 5G).

To further explore whether Rnf19a inhibits the inflammatory response through degradation of RIG-I, BV2 cells overexpressing Rnf19a were transfected with RIG-I N construct or empty vector, following JEV infection, and the expression of inflammatory cytokines was measured by qRT-PCR. We found that transfection of RIG-I N plasmid rescued the suppressive effect of Rnf19a on JEV-induced expression of inflammatory cytokines (Fig. 5H). These results suggest that Rnf19a inhibits the inflammatory response via mediating degradation of RIG-I during JEV infection.

To further verify the H3K27me3-Rnf19a-RIG-I axis regulates the inflammatory response in JEV-infected microglia, Rnf19a KD and NC BV2 cells were treated with EZH2 inhibitor, following JEV infection. The immunoblotting results showed that, Rnf19a expression was significantly upregulated while the protein abundance of RIG-I was significantly reduced in response to GSK343 treatment,  when Rnf19a was knockdown, RIG-I abundance was increased in both JEV- or mock-infected BV2 cells (Fig. 5I). Collectively, these results indicate that infection with JEV leads to a decrease in the expression of Rnf19a due to an increase in its H3K27me3 modification, which ultimately regulates the degradation of RIG-I.

## Discussion

Inflammation can have both beneficial and detrimental effects on the body. While it is typically a necessary immune response to fight pathogens and toxins, excessive inflammation can lead to harm. During JEV infection, the overactivation of inflammatory response in brain leads to neuro-damage. Therefore, understanding the molecular mechanism of JEV-induced excessive neuroinflammation is crucial for elucidating JEV pathogenesis. Our previous studies have demonstrated that JEV activates microglial cells through TLR-3/RIG-I-NF-κB/AP-1 signaling pathway [34], which leads to release of inflammatory cytokines, chemokines, ROS, and nitric oxide [14], and cause severe encephalitis. Recently, impressive studies revealed that histone methylation plays an important role in regulation the inflammatory response. It has been shown that H3K9 methylation and DNA methylation work together to silence TNF-α expression in endotoxin tolerance [35]. Additionally, reducing the expression of SET7/9 of H3K4 methyltransferase has been reported to decrease the expression of TNF-α-induced gene subsets like MCP-1, TNF-α and IL-8 in THP-1 macrophages [36]. However, it is currently unknown if histone methylation plays a role in regulating the inflammatory response during JEV infection. In this study, we demonstrated for the first time that the H3K27me3 modification is implicated in the neuroinflammatory response following JEV infection.

The presence of high level of H3K27me3 can not only promote the differentiation and proliferation of neurons, astrocytes and oligodendrocytes cells [37], but also contribute to inflammation of microglia [29]. Based on this, we speculated that the upregulation of H3K27me3 during JEV infection may be also linked to the occurrence of neuroinflammation. To test this hypothesis, we attempted to inhibit the H3K27me3 level in BV2 cells by either knocking down EZH2 or treating cells with the EZH2 inhibitor GSK343. As expected, interfering with EZH2 inhibited H3K27me3 modification and downregulated the inflammatory response in microglial cells during JEV infection. However, our findings suggest that JEV infection only results in a minor increase in EZH2 expression. This implies that the elevation of H3K27me3 levels following JEV infection is not primarily caused by the upregulation of EZH2 expression. Instead, it may occur through the recruitment of more EZH2 to histone H3 in an alternative manner. It has been known that the regulation of H3K27me3 involves the synergistic action of both histone methyltransferase and demethylases. Research has shown that secondary organ metastases display a notable increase in H3K27me3 levels compared to primary tumors. However, this increase is not linked to the EZH2 levels on these metastatic sites but instead is attributed to the crucial role played by demethylases like JMJD3/KDM6A in the process [38]. Here the upregulation of H3K27me3 caused by JEV may also be contributed by the downregulation of histone demethylases.

To identify genes that are transcriptionally suppressed by H3K27me3, we conducted a ChIP-seq analysis. This allowed us to screen for H3K27me3-inhibited genes. Rnf19a, a member of RING-in-between-RING (RBR) E3 ligases which has been reported to be involved in inflammation regulation, cancers, spermiogenesis, neurogenesis, synaptic plasticity and contextual fear memory [39–42], was selected for further study. Previous research has shown that Rnf19a can inhibit the activation of the downstream NF-κB signaling and thus reduce pro-inflammatory cytokine production by mediating the ubiquitination and degradation of TRAF6 [33]. However, our findings suggest that Rnf19a modulates inflammation in a different way. Specifically, we observed that Rnf19a mediates the ubiquitination and degradation of RIG-I, which inhibits the activation of the NF-κB signaling pathway. This difference in results may be due to the distinct cell type used in our study. Therefore, the study has identified a new mechanism for Rnf19a in regulating inflammatory response, although further research is required to determine how Rnf19a mediates ubiquitination of RIG-I.

Our research has also observed an intriguing phenomenon that Rnf19a is capable of degrading RIG-I through both the proteasome and lysosome pathways. Previous reports have indicated that ubiquitin ligases, such as Nedd4, can also facilitate protein degradation through the endosomal–lysosomal pathway [43]. Additionally, RNF152, a member of the RING Finger protein family like Rnf19a, has been linked to involvement in the lysosomal pathway [44]. Ubiquitins can be covalently linked through K48 or K11 residues, leading to the degradation of target proteins via the proteasome pathway. On the other hand, ubiquitins can attach chains through K63 residues, which can target proteins for lysosomal degradation [45]. Rnf19a may lead to different degradation pathways of RIG-I by connecting ubiquitin to different sites, but the specific mechanism requires further exploration.

RIG-I, as a cellular pattern recognition receptor, has been known to play a decisive role in recognizing genomic RNAs of flavivirus and thus inducing the production of interferon which may restrict the viral replication [46]. However, in this study, knockdown of EZH2 or Rnf19a did not show any impact on JEV replication in BV2 cells. The reason for this could be attributed to the inhibitory effect of JEV on downstream signal of type I interferon [47, 48], or the time delay of its antiviral effect. Additionally, while the N-terminal of RIG-I can partially rescue the downregulation of inflammatory factors caused by Rnf19a, its effectiveness was not as pronounced as anticipated in BV2 cells infected by JEV. We speculate that this may be due to the relatively low transfection efficiency of BV2 cells.

## Conclusions

This study highlights the potential for regulating the neuroinflammatory response caused by JEV infection through H3K27me3 modification in microglial cells. The research also found that increased H3K27me3 modification during JEV infection leads to downregulation of Rnf19a, which acts as a negative regulator of inflammation by degrading RIG-I. These findings provided a fresh perspective on the mechanism behind JEV-induced overactivation of neuroinflammation, viewed through an epigenetic lens, and suggest a potential therapeutic target for Japanese encephalitis.

## Supplementary Information

**Additional file 1: Figure S1.** JEV multiplication kinetics in BV2 cells, primary microglial cells and BALB/c mouse brains. BV2 cells (A) and primary mouse microglial cells (B) were infected with JEV at an MOI of 5. At 6, 12, 18, 24 and 30 hpi, viral titers were measured by plaque assay. (C) 6-week-old BALB/c mice (n = 9) were intracerebrally injected with 200 PFU of JEV P3 strain in 20 μL DMEM or equal amount of DMEM (mock infection). Mouse brain tissues were collected at 1, 3 and 5 dpi and subjected to plaque assay. Each dot represents a single mouse.

**Additional file 2: Figure S2.** JEV productively infects cortex and hypothalamus regions in mouse brain. 6-week-old BALB/c mice (n = 9) were intracerebrally injected with 200 PFU of JEV P3 strain in 20 μL DMEM or equal amount of DMEM (mock infection). Mice brain tissues were collected at 1, 3 and 5 dpi and subjected to IHC assay by using anti-JEV E monoclonal antibody. JEV E positive cells appear red while nucleus is purple. Scale bar, 200 μm.

**Additional file 3: Figure S3.** Cell viability determination of EZH2 knockdown and Rnf19a knockdown BV2 cells.

**Additional file 4: Figure S4.** Effect of EZH2 and Rnf19a knockdown on production of pro-inflammatory cytokines in supernatant of JEV-infected cells. EZH2 (A) or Rnf19a (B) knockdown (KD) or negative control (NC) BV2 cells were infected with JEV at an MOI of 5. The cell-supernatant was harvested at 36 hpi and the concentrations of TNF-α, CCL5 and IL-6 were determined by ELISA kits. Data are expressed as means ± SEM from three independent experiments. * *p* < 0.05, ** *p* < 0.01, *** *p* < 0.001, **** *p* < 0.0001.

**Additional file 5: Figure S5.** Effect of EZH2 knockdown, GSK343 and Rnf19a knockdown on JEV replication in BV2 cells and primary mouse microglial cells. (A) EZH2 KD and NC BV2 cells were infected with JEV at an MOI of 5. The viral titers were measured by plaque assay at 6, 12, 18, 24 and 30 hpi. (B) Primary microglial cells were treated with GSK343 (10 μM) following JEV infection at an MOI of 5. The viral titers were measured by plaque assay at 6, 12, 18, 24 and 30 hpi. (C) Rnf19a KD and NC BV2 cells were infected with JEV at an MOI of 5. The viral titers were measured by plaque assay at 6, 12, 18, 24 and 30 hpi.

**Additional file 6: Figure S6.** Cell viability determination of GSK343-treated in BV2 cells (A) and primary mouse microglial cells (B).

**Additional file 7:** S7.

## Data Availability

The ChIP-sequencing data are available on BioProject repository (accession number: PRJNA899838).

## Abbreviations

JEV: Japanese encephalitis virus
H3K27me3: Tri-methylation of histone H3 lysine 27
ChIP-sequencing: Chromatin immunoprecipitation sequencing
RIG-I: Retinoic acid-inducible gene I
Rnf19a: Ring finger protein 19A
h.p.i: Hours post-infection
d.p.i: Days post-infection
PFU: Plaque-forming units
MOI: Multiplicity of infection

## References

[1] Solomon T (2006). Control of Japanese encephalitis–within our grasp?. N Engl J Med.

[2] Yun SI, Lee YM (2014). Japanese encephalitis The virus and vaccines. Hum Vaccin Immunother.

[3] Thongtan T, Cheepsunthorn P, Chaiworakul V, Rattanarungsan C, Wikan N, Smith DR (2010). Highly permissive infection of microglial cells by Japanese encephalitis virus: a possible role as a viral reservoir. Microbes Infect.

[4] Ghoshal A, Das S, Ghosh S, Mishra MK, Sharma V, Koli P, Sen E, Basu A (2007). Proinflammatory mediators released by activated microglia induces neuronal death in Japanese Encephalitis. Glia.

[5] Stoll G, Jander S (1999). The role of microglia and macrophages in the pathophysiology of the CNS. Prog Neurobiol.

[6] Prinz M, Mildner A (2011). Microglia in the CNS: immigrants from another world. Glia.

[7] Unni SK, Ruzek D, Chhatbar C, Mishra R, Johri MK, Singh SK (2011). Japanese encephalitis virus: from genome to infectome. Microbes Infect.

[8] Arifuzzaman S, Das A, Kim SH, Yoon T, Lee YS, Jung KH, Chai YG (2017). Selective inhibition of EZH2 by a small molecule inhibitor regulates microglial gene expression essential for inflammation. Biochem Pharmacol.

[9] Gonzalez-Scarano F, Baltuch G (1999). Microglia as mediators of inflammatory and degenerative diseases. Annu Rev Neurosci.

[10] Quagliarello VJ, Wispelwey B, Long WJ, Scheld WM (1991). Recombinant human interleukin-1 induces meningitis and blood-brain-barrier injury in the rat—characterization and comparison with tumor-necrosis-factor. J Clin Investig.

[11] Ghosh D, Basu A (2009). Japanese encephalitis—a pathological and clinical perspective. Plos Negl Trop Dis.

[12] Thounaojam MC, Kaushik DK, Kundu K, Basu A (2014). MicroRNA-29b modulates Japanese encephalitis virus-induced microglia activation by targeting tumor necrosis factor alpha-induced protein 3. J Neurochem.

[13] Hazra B, Chakraborty S, Bhaskar M, Mukherjee S, Mahadevan A, Basu A (2019). miR-301a regulates inflammatory response to Japanese encephalitis virus infection via suppression of NKRF activity. J Immunol.

[14] Chen CJ, Ou YC, Chang CY, Pan HC, Liao SL, Chen SY, Raung SL, Lai CY (2012). Glutamate released by Japanese encephalitis virus-infected microglia involves TNF-alpha signaling and contributes to neuronal death. Glia.

[15] Ashraf U, Zhu B, Ye J, Wan S, Nie Y, Chen Z, Cui M, Wang C, Duan X, Zhang H (2016). MicroRNA-19b-3p modulates Japanese encephalitis virus-mediated inflammation via targeting RNF11. J Virol.

[16] Cheray M, Joseph B. Epigenetics control microglia plasticity. Front Cell Neurosci. 2018; 12. 10.3389/fncel.2018.00243 PMC6085560 30123114

[17] Thomas EA (2017). Histone posttranslational modifications in schizophrenia. Neuroepigenomics Aging Dis.

[18] Audia JE, Campbell RM (2016). Histone modifications and cancer. Cold Spring Harb Perspect Biol.

[19] Milavetz BI, Balakrishnan L (2015). Viral epigenetics. Methods Mol Biol.

[20] Stallcup MR (2001). Role of protein methylation in chromatin remodeling and transcriptional regulation. Oncogene.

[21] Martin C, Zhang Y (2005). The diverse functions of histone lysine methylation. Nat Rev Mol Cell Biol.

[22] Zeng J, Chen B (2014). Epigenetic mechanisms in the pathogenesis of diabetic retinopathy. Ophthalmologica.

[23] Black JC, Van Rechem C, Whetstine JR (2012). Histone lysine methylation dynamics: establishment, regulation, and biological impact. Mol Cell.

[24] Margueron R, Justin N, Ohno K, Sharpe ML, Son J, Drury WJ, Voigt P, Martin SR, Taylor WR, De Marco V (2009). Role of the polycomb protein EED in the propagation of repressive histone marks. Nature.

[25] Zhou J, Huang S, Wang ZY, Huang JN, Xu L, Tang XF, Wan YSY, Li QJ, Symonds ALJ, Long HX, Zhu B. Targeting EZH2 histone methyltransferase activity alleviates experimental intestinal inflammation. Nat Commun. 2019; 10. 10.1038/s41467-019-10176-2 PMC6547712 31160593

[26] Hui TQ, Peng A, Zhao Y, Yang J, Ye L, Wang CL (2018). EZH2 regulates dental pulp inflammation by direct effect on inflammatory factors. Arch Oral Biol.

[27] Tan J, Yang XJ, Zhuang L, Jiang X, Chen W, Lee PL, Karuturi RKM, Tan PBO, Liu ET, Yu Q (2007). Pharmacologic disruption of polycomb-repressive complex 2-mediated gene repression selectively induces apoptosis in cancer cells. Genes Dev.

[28] Turgeon N, Blais M, Delabre JF, Asselin C (2013). The histone H3K27 methylation mark regulates intestinal epithelial cell density-dependent proliferation and the inflammatory response. J Cell Biochem.

[29] Zhang XL, Wang Y, Yuan J, Li N, Pei SY, Xu J, Luo X, Mao CM, Liu JL, Yu T (2018). Macrophage/microglial Ezh2 facilitates autoimmune inflammation through inhibition of Socs3. J Exp Med.

[30] Thounaojam MC, Kundu K, Kaushik DK, Swaroop S, Mahadevan A, Shankar SK, Basu A (2014). MicroRNA 155 regulates Japanese encephalitis virus-induced inflammatory response by targeting Src homology 2-containing inositol phosphatase 1. J Virol.

[31] Chen Z, Wang X, Ashraf U, Zheng B, Ye J, Zhou D, Zhang H, Song Y, Chen H, Zhao S, Cao S (2018). Activation of neuronal N-methyl-D-aspartate receptor plays a pivotal role in Japanese encephalitis virus-induced neuronal cell damage. J Neuroinflammation.

[32] Cao R, Wang L, Wang H, Xia L, Erdjument-Bromage H, Tempst P, Jones RS, Zhang Y (2002). Role of histone H3 lysine 27 methylation in Polycomb-group silencing. Science.

[33] Wu C, Su Z, Lin M, Ou J, Zhao W, Cui J, Wang RF (1977). NLRP11 attenuates Toll-like receptor signalling by targeting TRAF6 for degradation via the ubiquitin ligase RNF19A. Nat Commun.

[34] Jiang R, Ye J, Zhu B, Song Y, Chen H, Cao S (2014). Roles of TLR3 and RIG-I in mediating the inflammatory response in mouse microglia following Japanese encephalitis virus infection. J Immunol Res.

[35] El Gazzar M, Yoza BK, Chen X, Hu J, Hawkins GA, McCall CE (2008). G9a and HP1 couple histone and DNA methylation to TNFalpha transcription silencing during endotoxin tolerance. J Biol Chem.

[36] Li Y, Reddy MA, Miao F, Shanmugam N, Yee JK, Hawkins D, Ren B, Natarajan R (2008). Role of the histone H3 lysine 4 methyltransferase, SET7/9, in the regulation of NF-kappaB-dependent inflammatory genes. Relevance to diabetes and inflammation. J Biol Chem.

[37] Pereira JD, Sansom SN, Smith J, Dobenecker MW, Tarakhovsky A, Livesey FJ (2010). Ezh2, the histone methyltransferase of PRC2, regulates the balance between self-renewal and differentiation in the cerebral cortex. Proc Natl Acad Sci USA.

[38] Verma A, Singh A, Singh MP, Nengroo MA, Saini KK, Satrusal SR, Khan MA, Chaturvedi P, Sinha A, Meena S (2022). EZH2-H3K27me3 mediated KRT14 upregulation promotes TNBC peritoneal metastasis. Nat Commun.

[39] Zhu Q, Huang JZ, Huang HY, Li H, Yi PQ, Kloeber JA, Yuan J, Chen YP, Deng M, Luo KT, et al. RNF19A-mediated ubiquitination of BARD1 prevents BRCA1/BARD1-dependent homologous recombination. Nat Commun. 2021; 12. 10.1038/s41467-021-27048-3 PMC8599684 34789768

[40] Cheng Y, Hu YJ, Wang HX, Zhao Z, Jiang XZ, Zhang Y, Zhang JM, Tong Y, Qiu XS (2021). Ring finger protein 19A is overexpressed in non-small cell lung cancer and mediates p53 ubiquitin-degradation to promote cancer growth. J Cell Mol Med.

[41] Rivkin E, Kierszenbaum AL, Gil M, Tres LL (2009). Rnf19a, a ubiquitin protein ligase, and Psmc3, a component of the 26S proteasome, tether to the acrosome membranes and the head-tail coupling apparatus during rat spermatid development. Dev Dyn.

[42] Park H, Yang J, Kim R, Li Y, Lee Y, Lee C, Park J, Lee D, Kim H, Kim E. Mice lacking the PSD-95-interacting E3 ligase, Dorfin/Rnf19a, display reduced adult neurogenesis, enhanced long-term potentiation, and impaired contextual fear conditioning. Sci Rep. 2015; 5. 10.1038/srep16410 PMC4639748 26553645

[43] Tofaris GK, Kim HT, Hourez R, Jung JW, Kim KP, Goldberg AL (2011). Ubiquitin ligase Nedd4 promotes alpha-synuclein degradation by the endosomal-lysosomal pathway. Proc Natl Acad Sci U S A.

[44] Zhang S, Wu W, Wu Y, Zheng J, Suo T, Tang H, Tang J (2010). RNF152, a novel lysosome localized E3 ligase with pro-apoptotic activities. Protein Cell.

[45] Pickart CM, Fushman D (2004). Polyubiquitin chains: polymeric protein signals. Curr Opin Chem Biol.

[46] Guo HY, Zhang XC, Jia RY (2018). Toll-like receptors and RIG-I-like receptors play important roles in resisting flavivirus. J Immunol Res.

[47] Ye J, Chen Z, Li Y, Zhao Z, He W, Zohaib A, Song Y, Deng C, Zhang B, Chen H, Cao S. Japanese encephalitis virus NS5 inhibits type I interferon (IFN) production by blocking the nuclear translocation of IFN regulatory factor 3 and NF-kappaB. J Virol 2017; 91. 10.1128/JVI.00039-17 PMC5375679 28179530

[48] Li Q, Zhou D, Jia F, Zhang L, Ashraf U, Li Y, Duan H, Song Y, Chen H, Cao S, Ye J: Japanese encephalitis virus NS1’ protein interacts with host CDK1 protein to regulate antiviral response. Microbiol Spectr. 2021:e0166121. 10.1128/Spectrum.01661-21 PMC8579942 34756071

