# Supplementary figures and images for: H3K27me3 of Rnf19a promotes neuroinflammatory response during Japanese encephalitis virus infection
Source: J Neuroinflammation. 2023 Jul 21;20:168. doi: 10.1186/s12974-023-02852-4 (PMC10362728; doi:10.1186/s12974-023-02852-4)

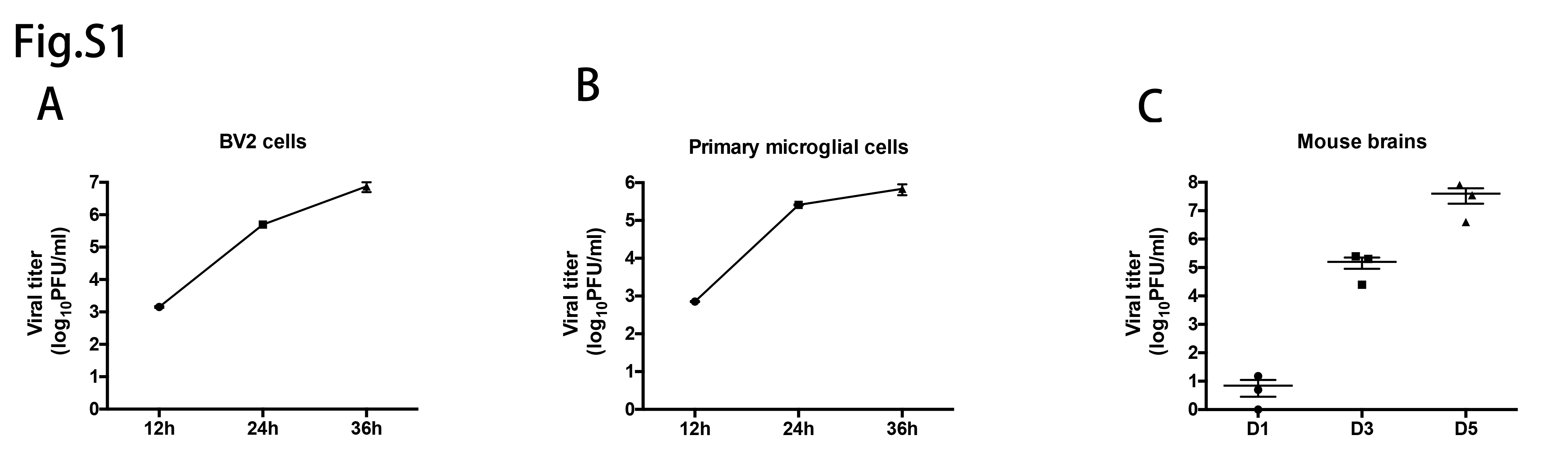

Supplement: Supplementary file 1 — Additional file 1: Figure S1. JEV multiplication kinetics in BV2 cells, primary microglial cells and BALB/c mouse brains. BV2 cells (A) and primary mouse microglial cells (B) were infected with JEV at an MOI of 5. At 6, 12, 18, 24 and 30 hpi, viral titers were measured by plaque assay. (C) 6-week-old BALB/c mice (n = 9) were intracerebrally injected with 200 PFU of JEV P3 strain in 20 μL DMEM or equal amount of DMEM (mock infection). Mouse brain tissues were collected at 1, 3 and 5 dpi and subjected to plaque assay. Each dot represents a single mouse. [file 12974_2023_2852_MOESM1_ESM.png]

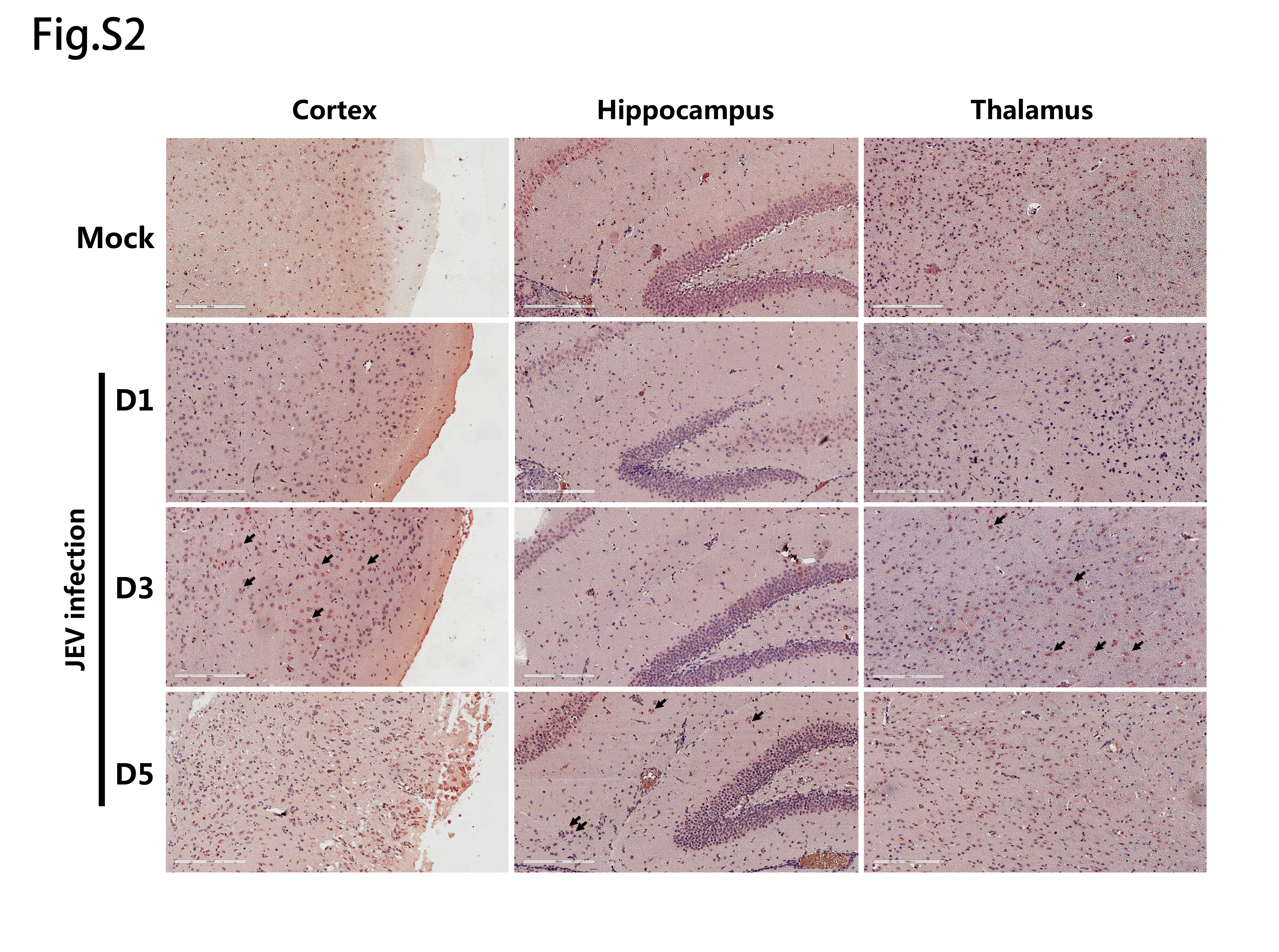

Supplement: Supplementary file 2 — Additional file 2: Figure S2. JEV productively infects cortex and hypothalamus regions in mouse brain. 6-week-old BALB/c mice (n = 9) were intracerebrally injected with 200 PFU of JEV P3 strain in 20 μL DMEM or equal amount of DMEM (mock infection). Mice brain tissues were collected at 1, 3 and 5 dpi and subjected to IHC assay by using anti-JEV E monoclonal antibody. JEV E positive cells appear red while nucleus is purple. Scale bar, 200 μm. [file 12974_2023_2852_MOESM2_ESM.png]

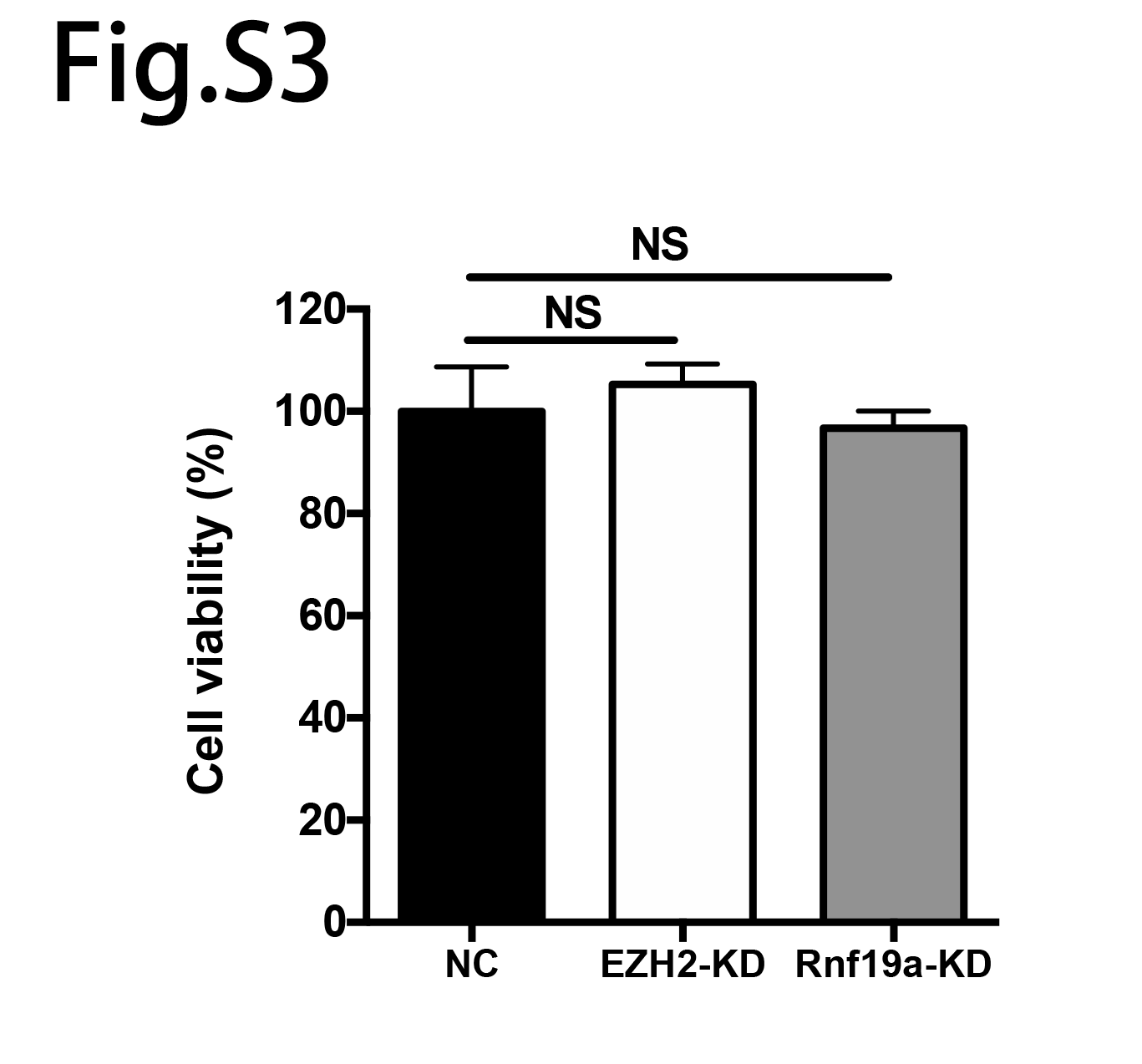

Supplement: Supplementary file 3 — Additional file 3: Figure S3. Cell viability determination of EZH2 knockdown and Rnf19a knockdown BV2 cells. [file 12974_2023_2852_MOESM3_ESM.png]

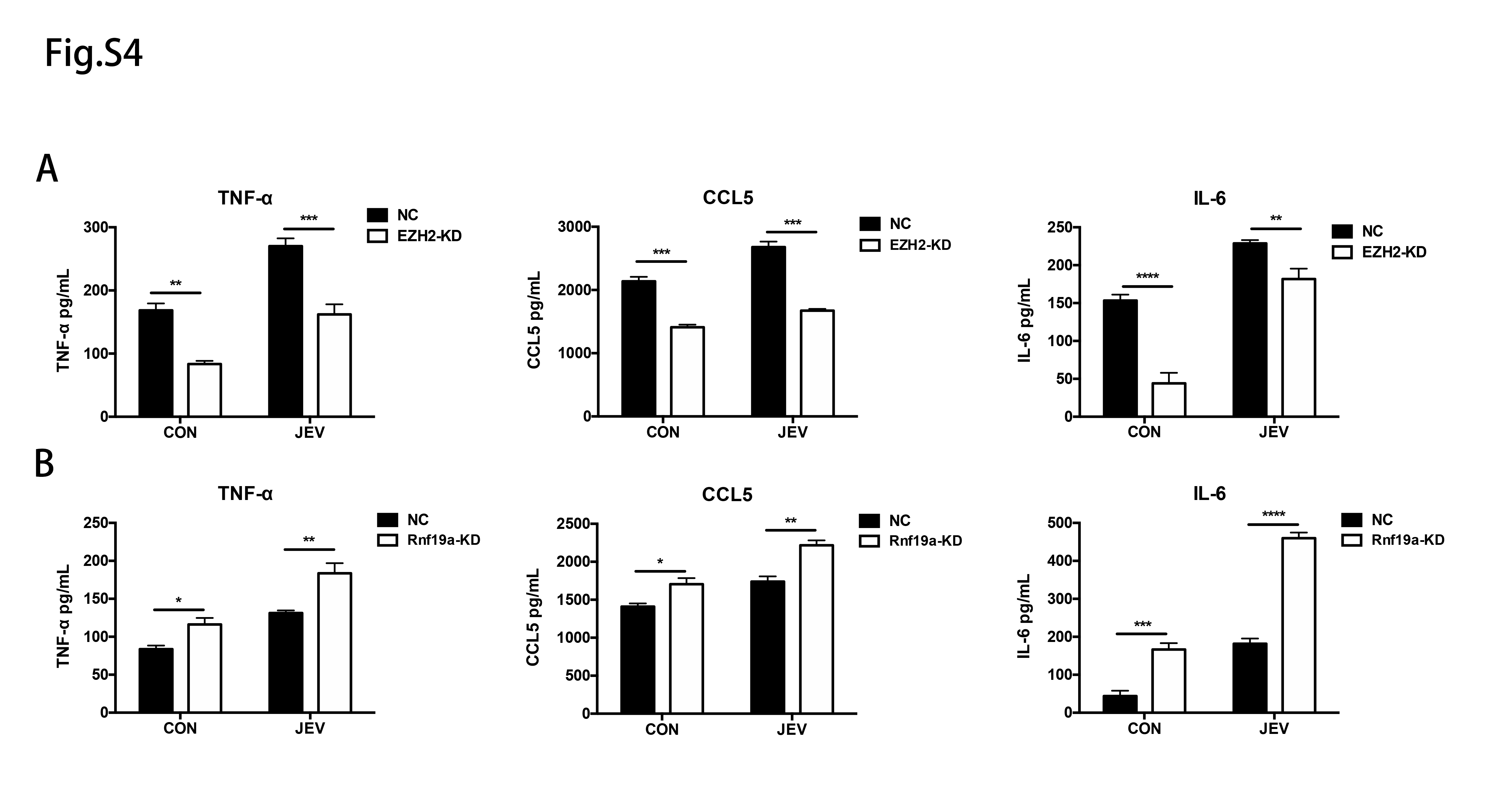

Supplement: Supplementary file 4 — Additional file 4: Figure S4. Effect of EZH2 and Rnf19a knockdown on production of pro-inflammatory cytokines in supernatant of JEV-infected cells. EZH2 (A) or Rnf19a (B) knockdown (KD) or negative control (NC) BV2 cells were infected with JEV at an MOI of 5. The cell-supernatant was harvested at 36 hpi and the concentrations of TNF-α, CCL5 and IL-6 were determined by ELISA kits. Data are expressed as means ± SEM from three independent experiments. * p < 0.05, ** p < 0.01, *** p < 0.001, **** p < 0.0001. [file 12974_2023_2852_MOESM4_ESM.png]

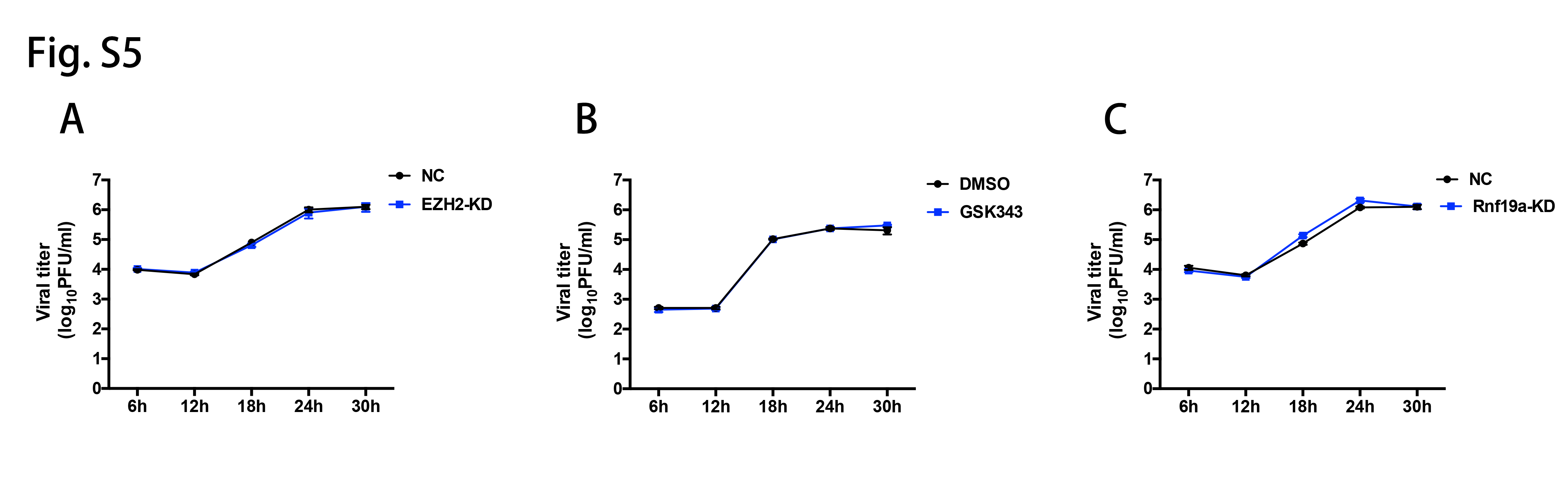

Supplement: Supplementary file 5 — Additional file 5: Figure S5. Effect of EZH2 knockdown, GSK343 and Rnf19a knockdown on JEV replication in BV2 cells and primary mouse microglial cells. (A) EZH2 KD and NC BV2 cells were infected with JEV at an MOI of 5. The viral titers were measured by plaque assay at 6, 12, 18, 24 and 30 hpi. (B) Primary microglial cells were treated with GSK343 (10 μM) following JEV infection at an MOI of 5. The viral titers were measured by plaque assay at 6, 12, 18, 24 and 30 hpi. (C) Rnf19a KD and NC BV2 cells were infected with JEV at an MOI of 5. The viral titers were measured by plaque assay at 6, 12, 18, 24 and 30 hpi. [file 12974_2023_2852_MOESM5_ESM.png]

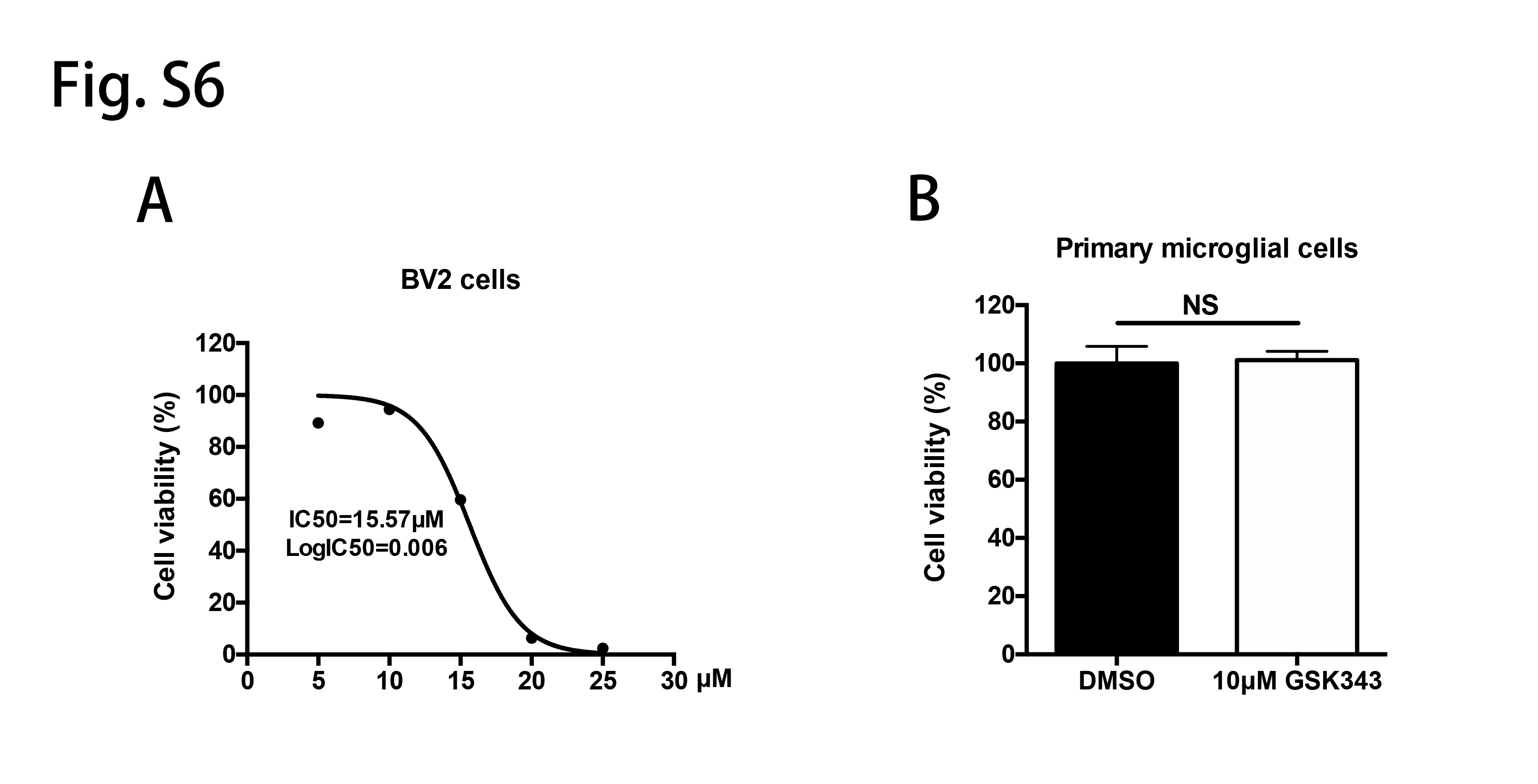

Supplement: Supplementary file 6 — Additional file 6: Figure S6. Cell viability determination of GSK343-treated in BV2 cells (A) and primary mouse microglial cells (B). [file 12974_2023_2852_MOESM6_ESM.png]
